# The Limitless Future of RNA Therapeutics

**DOI:** 10.3389/fbioe.2021.628137

**Published:** 2021-03-18

**Authors:** Tulsi Ram Damase, Roman Sukhovershin, Christian Boada, Francesca Taraballi, Roderic I. Pettigrew, John P. Cooke

**Affiliations:** ^1^RNA Therapeutics Program, Department of Cardiovascular Sciences, Houston Methodist Research Institute, Houston, TX, United States; ^2^Colleges of Medicine, Engineering, Texas A&M University and Houston Methodist Hospital, Houston, TX, United States; ^3^Center for Musculoskeletal Regeneration, Houston Methodist Research Institute, Houston, TX, United States; ^4^Department of Orthopedics and Sports Medicine, Houston Methodist Hospital, Houston, TX, United States

**Keywords:** RNA therapeutics, delivery of RNA therapeutics, hospital-based RNA therapeutics, messenger RNAs (mRNAs), self-amplifying mRNA

## Abstract

Recent advances in the generation, purification and cellular delivery of RNA have enabled development of RNA-based therapeutics for a broad array of applications. RNA therapeutics comprise a rapidly expanding category of drugs that will change the standard of care for many diseases and actualize personalized medicine. These drugs are cost effective, relatively simple to manufacture, and can target previously undruggable pathways. It is a disruptive therapeutic technology, as small biotech startups, as well as academic groups, can rapidly develop new and personalized RNA constructs. In this review we discuss general concepts of different classes of RNA-based therapeutics, including antisense oligonucleotides, aptamers, small interfering RNAs, microRNAs, and messenger RNA. Furthermore, we provide an overview of the RNA-based therapies that are currently being evaluated in clinical trials or have already received regulatory approval. The challenges and advantages associated with use of RNA-based drugs are also discussed along with various approaches for RNA delivery. In addition, we introduce a new concept of hospital-based RNA therapeutics and share our experience with establishing such a platform at Houston Methodist Hospital.

## Introduction

RNA Therapeutics comprise a rapidly expanding category of drugs that will speed solutions to the clinic; will actualize personalized medicine; and will make the term “undruggable” obsolete. The first RNA drugs have been approved, and many more are in development. We are in the midst of a therapeutic revolution, the likes of which have not been seen since the advent of recombinant protein technology almost 50 years ago in Silicon Valley. Accordingly, we will review recent developments in RNA Therapeutics, and their promise to alter the landscape of the pharmaceutical industry.

Conventional drug strategy relies on the ability of small molecule drugs to target active sites of proteins so as to inhibit or alter their function. It is well known that only ∼1.5% of the human genome encodes proteins (Ezkurdia et al., 2014). Furthermore, only 10–14% of proteins have active binding sites that are “druggable” targets for small molecules (Hopkins and Groom, 2002). Thus the “druggable” targets for small molecule therapies is limited. This limitation was addressed in part by the revolution of recombinant protein technology which had its beginnings in Silicon Valley in the late 1970’s (Russo, 2003). The development of genetic engineering by Stanley Cohen at Stanford University and Herb Boyer at UCSF provided the platform for generating recombinant proteins. Recombinant protein technology has become a significant share of the pharmaceutical market. In 2019, the FDA Center for Drug Evaluation and Research (CDER) approved 48 new agents, of which 10 were recombinant proteins, peptides or drug-antibody conjugates (Mullard, 2020). The Center for Biologics Evaluation and Research (CBER) is primarily responsible for evaluation and approval of vaccines, allergenic products, blood and blood products, plasma derivatives, cellular and gene therapy products. In 2019, CBER approved 22 new applications, one of which was a recombinant protein, three were attenuated virus vaccines, and one was a DNA therapeutic, Zolgensma (FDA, 2019). Recombinant proteins have limitations as drugs, particularly due to size and stability issues (Antosova et al., 2009; Lam et al., 2015). Furthermore, they must be properly folded and often require post-translational modifications (Li et al., 2015) that complicate the synthetic process. By contrast, nucleic-acid based strategies avoid many of these limitations as they make use of the translational machinery of the mammalian cell. Because DNA therapeutics pre-dated RNA therapeutics, a brief discussion of these nucleic-acid based cousins of RNA is useful for historical and comparative purposes.

## DNA Therapeutics

DNA drugs generate therapeutic proteins when delivered to the nuclei of the patient’s cells. DNA drugs may be delivered as plasmids or integrated into a viral vector.

### DNA Plasmids

DNA plasmids are high molecular weight, double-stranded (ds), circular DNA molecules that encode a therapeutic protein. Such proteins could replace defective or missing proteins in the patient (Saraswat et al., 2009). DNA plasmids can be used in: (1) gene therapy, (2) vaccination, and (3) cell therapy. The plasmid DNA (pDNA) must penetrate the cytoplasmic and nuclear membranes to gain access to the nucleus. In the nucleus, the pDNA is transcribed into mRNA that encodes the desired protein in the patient’s body. As an example, the pDNA drug VM202 is a 7377 base pair plasmid DNA gene therapy that encodes both isoforms of human hepatocyte growth factor, HGF (Kessler et al., 2015; ClinicalTrials.gov, 2020d). This drug is in a Phase III clinical trial to assess its benefit in treating painful diabetic peripheral neuropathy (DPN).

### Viral Vectors

DNA-based drugs may be directed toward replacing defective or missing protein(s) (Schmeer et al., 2017; Daley, 2019). However, concerns regarding integration of foreign DNA into the host chromosomes and disruption of normal gene function has led to a search for non-integrating strategies. Adeno-Associated virus (AAV) is a common viral vector for the delivery of DNA encoding a therapeutic protein with minimal risk for integration. The AAV is a small (25 nm) icosahedral human parvovirus that contains a linear single-stranded DNA (4.7 kb). After removing key AAV viral genes, the desired gene is inserted into the AAV DNA for expression. The AAV vector delivers the desired gene to the target cell, for example to restore normal protein function (Tratschin et al., 1984; Naso et al., 2017). Alternatively, the AAV vector can be utilized to deliver interference RNA to downregulate the expression of a specific gene. Tomar et al. (2003) demonstrated the use of AAV to express hairpin siRNA in HeLa S3 cells to downregulate expression of caspase 8 and p53.

The Chinese food and drug regulatory agency approved the first DNA therapy drug, Gendicine, in 2003 to treat head and neck squamous cancer. Gendicine is an adenoviral vector encoding the wild type (wt) p53 gene which restores the expression of this tumor suppressor function to treat cancer (Zhang et al., 2018). Approximately 60–80% of all cancers manifest a p53 deficiency. In 2012, Glybera became the first DNA therapy to be approved in Europe. Glybera was an adeno-associated virus (serotype 1) encoding the lipoprotein lipase (LPL) gene to reverse LPL deficiency. This is a rare genetic disease that increases the levels of fat in the blood and cause severe pancreatitis. Glybera was not commercially successful and is no longer available (Bryant et al., 2013). Luxturna is an AAV (serotype 2) therapy encoding the RPE65 gene which received FDA approval in December 2017 and European approval in 2018. It is indicated for patients for RPE65-mediated inherited retinal dystrophy and improves vision by restoring RPE65 protein levels (Maguire et al., 2019). Zolgensma is an AAV vector-based gene therapy that delivers a fully functional copy of the human SMN gene into the target motor neuron cells. A one-time intravenous administration results in expression of the SMN protein in a child’s motor neurons, which improves muscle movement and function and survival of children with spinal muscular atrophy, SMA (Mahajan, 2019).

DNA-based vaccines encode specific antigen(s) to induce a protective immune response (Daley, 2019). Imlygic is a genetically modified therapy approved in United States, China, Europe, and Australia to treat malignant tumors. In this DNA therapy, the ICP34.5 gene is deleted to attenuate the natural herpes simplex virus type 1, HSV-1, which diminishes infection of normal tissues, but which enhances preferential tumor killing. The drug inserts into tumor cells, replicating and expressing a protein which evokes a cytotoxic immune response to the cancer cells (Conry et al., 2018; Amgen, 2020). DNA-based vaccines may also target infectious agents. For example, Innovio has developed a DNA vaccine encoding the spike protein of SARS-CoV-2 for intramuscular injection to induce immunity to the virus (INOVIO, 2020). Theirs was the first DNA vaccine to enter clinical trials for COVID-19. Other DNA vaccine developers include Genexine Consortium; Osaka University with Takara Bio, Anges & Cytiva; and Zydus Cadila Healthcare Limited (CovidVax, 2020; Milkeninstitute, 2020).

DNA vectors can be used to generate novel cell therapies. Typically, the therapeutic DNA is transfected into the cells *ex vivo* to alter cell phenotype or function, and then these cells are expanded and delivered into the patient (Daley, 2019). Kymriah is CD19-targeting CAR T cells immunotherapy to treat leukemia, lymphoma, and pediatric cancer in a single dose (approved in United States and Europe in 2017 and 2018 respectively). Enriched T cells from patient’s peripheral blood mononuclear cells are transduced with a lentiviral vector encoding a chimeric antigen receptor (CAR) against CD19. Subsequently, the transduced T cells are expanded, formulated into a suspension, and cryopreserved for later delivery (Zheng et al., 2018; FDA, 2020). Yescarta is another CD19-targeting CAR immunotherapy to treat large B cell lymphoma. Yescarta uses a retroviral vector to insert the CD-19 specific CAR into T cells. The anti-CD19 CAR T-cells are infused back into the patient’s body to kill CD19-expressing target cells. This drug was approved in the United States (2017) and Europe (2018) (Fala, 2018; Zheng et al., 2018).

Strimvelis is a personalized DNA-based medicine approved in Europe in 2016 to treat patients with a very rare disease called Severe Combined Immunodeficiency due to Adenosine Deaminase deficiency (ADA-SCID). Strimvelis uses the patient’s own CD34^+^ cells generated from hematopoietic stem cell (HSCs). The CD34^+^ cells are then transduced with a gammaretrovirus vector carrying the gene for human adenosine deaminase (ADA), then reinfused into the patient. These cells home to the patient’s bone marrow where CD34^+^ cells replicate and generate normal ADA protein to correct the deficiency (South et al., 2018). Zyntelgo was approved in Europe in 2019 for the treatment of beta-thalassemia. Zyntelgo comprises a lentiviral vector to introduce the beta globin gene into autologous blood-derived CD34^+^ cells *ex vivo*. The genetically modified cells are then re-infused and home to the patient’s bone marrow to generate red blood cells with normally functioning hemoglobin (Schuessler-Lenz et al., 2020). A similar approach was taken by investigators at St. Jude Children’s Research Hospital, to treat severe combined immunodeficiency (SCID), also called bubble boy disease. This rare- life-threatening disorder is caused by mutations in the gene that encodes the common γ-chain (IL2RG), which is shared by multiple cytokine receptors. This protein is necessary for the development and function of lymphocytes, so children with this disease lack normal immune function. The St. Jude investigators used a lentiviral vector to transfect autologous blood-derived CD34^+^ cells *ex vivo* with the IL2RG gene, which cells are then re-infused (Stjude, 2020; St Jude Children’s Research Hospital, 2020). As described below, this gene therapy is effective at restoring immune function in SCID.

### CRISPR-Associated Protein 9 (CRISPR-Cas9)

Genome editing tools are used to add, remove or alter the genetic material at particular locations in the genome. There are several genome editing methods, as typified by clustered regularly interspaced short palindromic repeats and CRISPR-associated protein 9, CRISPR-Cas9 (Jiang and Doudna, 2017). Ishino et al. (1987) were first to discover the CRISPR DNA sequence. The CRISPR-Cas9 is a component of the bacterial adaptive immune system. In bacteria, two RNA molecules and the protein Cas9 bind to a foreign target. One of these molecules, called *trans*-activating CRISPR RNA (tracrRNA), serves as a scaffold and binds to Cas9, a DNA endonuclease. The other molecule, called CRISPR RNA (crRNA), has sequence homology to the foreign DNA (Jiang and Doudna, 2017; Karlgren et al., 2018; Zhan et al., 2019) and ensures cleavage specificity. This native immune defense has been modified for genome editing. In one version of the modified CRISPR-Cas9 technology, both RNA molecules are linked into a single guide RNA (sgRNA). The target DNA sequence is followed by a protospacer adjacent motif (PAM), also known as “NGG” sequence, which is a short (2–6 bp) DNA sequence. Cas9 cleaves the target DNA sequence 3 bases upstream of the PAM and creates a double-strand break. The latter can be repaired by two mechanisms: non-homologous end joining (NHEJ), and homology-directed repair (HDR). NHEJ is an error-prone process that introduces small deletions or insertions (indels) and disrupts the targeted locus (gene knock-out). HDR is a more precise process where a short donor DNA sequence is used for the double-stranded break repair (Jiang and Doudna, 2017; Karlgren et al., 2018), facilitating a gene knock-in.

EDIT-101 (Editas Medicine) is an early stage clinical CRISPR medicine to treat patients suffering from Leber Congenital Amaurosis type 10 (LCA10). LCA10 is a genetic blindness caused by an A to G point mutation within intron 26 in the CEP290 gene involved in phototransduction. This mutation results in a splicing defect to include a 128 bp cryptic exon in CEP290 transcript, thereby leading to expression of non-functional mutant protein. EDIT-101 is designed to deliver *Staph. aureus* Cas9 and two CEP290-specific guide RNAs to target cells by subretinal injection using an adeno-associated viral vector. In EDIT-101, Cas9 is driven by photoreceptor cell-specific promoter, rhodopsin kinase, and the resulting break in DNA is repaired by NHEJ, resulting in normal CEP290 expression and restoration of photoreceptor function (Maeder et al., 2019; Research and Pipeline – Editas, 2020).

A few other CRISPR-Cas9-based drugs are also in clinical trials now. For instance, CTX001, CTX110, CTX120, and CTX130 from CRISPR Therapeutics (Pipeline – CRISPR, 2019c). CTX001 is designed to treat both sickle cell disease and beta thalassemia. It disrupts the erythroid lineage-specific enhancer of the BCL11A gene in autologous blood stem cells and thus increases production of γ-globin, a component of fetal hemoglobin (HbF). The latter can serve as an alternative to the patient’s defective adult hemoglobin (HbA), and its increased level is observed in the patient’s blood cells after treatment with CTX001(Hemoglobinopathies – CRISPR, 2019a). CTX 110, CTX120, and CTX130 are being developed as immunotherapies, which, respectively, create allogenic CAR-T cells against CD19 for B-cell malignancies, BCMA for multiple myeloma, and CD70 for both hematologic cancers (certain lymphomas) and solid tumors (renal cell carcinoma) (Immuno-Oncology – CRISPR, 2019b).

All DNA-based drugs must penetrate two membranes, the cytoplasmic and the nuclear membranes, to have their effect. Because they enter the nucleus, DNA drugs raise safety concerns due to potential integration into the host genome (Ledwith et al., 2000). These limitations can be addressed by RNA therapeutics which only need to reach the cell cytoplasm, and which represents no risk of chromosomal integration (Lundstrom, 2018a; Shin et al., 2018).

## RNA Therapeutics

The development of RNA drugs has largely focused on two approaches: (1) antisense RNA (RNAi), where short oligonucleotides recognize and hybridize to complementary sequences in endogenous RNA transcripts and alter their processing (Stephenson and Zamecnik, 1978; Shen and Corey, 2018); and (2) message RNA (mRNA), where mRNAs encoding certain peptides or proteins elicit their transient expression in the cytoplasm (for instance, to replace defective proteins or present antigens for vaccination) (Wolff et al., 1990; Ulmer and Geall, 2016). The development of RNA therapeutics required that several major hurdles be overcome, specifically the (1) rapid degradation of exogenous RNA by RNases that are ubiquitous in the environment and tissues; (2) delivery of negatively charged RNA across hydrophobic cytoplasmic membrane; and (3) strong immunogenicity of exogenous RNA that caused cell toxicity and impaired translation into therapeutic proteins.

**FIGURE 1:**
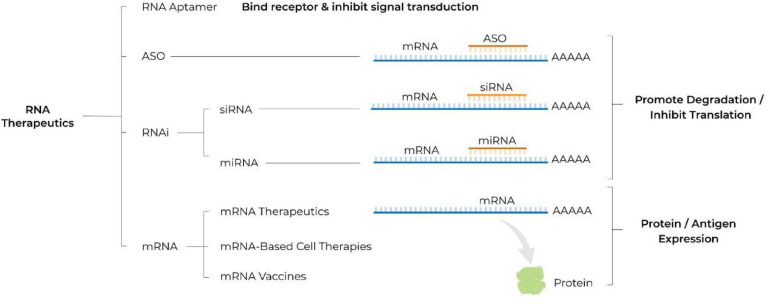
Schematic illustrating different classes of RNA therapeutics. ASO, antisense oligonucleotide; RNA, ribonucleic acid; RNAi, RNA interference; siRNA, small interfering RNA; miRNA, microRNA; mRNA, messenger RNA; A, adenosine molecule; AAAAA, poly A tail.

These hurdles have been substantially overcome with recent advancements in RNA biology, bioinformatics, separation science and nanotechnology thereby facilitating the recent rapid development of RNA therapeutics. Advantages of RNA-based drugs that are driving development include: (1) their ability to act on targets that are otherwise “undruggable” for a small molecule or a protein; (2) their rapid and cost effective development, by comparison to that of small molecules or recombinant proteins; (3) the ability to rapidly alter the sequence of the mRNA construct for personalized treatments or to adapt to an evolving pathogen. Below, we review the different classes of RNA therapeutics (see Figure 1), the challenges and advantages associated with their use, and provide an overview of the therapeutics under development as well as of those already available on the market (see Table 1). In addition, we introduce a new concept of hospital-based RNA therapeutics and share our experience with establishing such a platform at Houston Methodist Hospital.

**TABLE 1:** RNA therapeutics approved for clinical use or undergoing clinical trials.

Drug	Type of RNA	Company	Route of delivery	Condition/Disease	Status
Nusinersen (Spinraza)	ASO	Ionis	Intrathecal	Spinal muscular atrophy	FDA approval in 2016
Eteplirsen (Exondys 51)	ASO	Sarepta	Intravenous	Duchenne muscular dystrophy	FDA approval in 2016
Inotersen (Tegsedi)	ASO	Ionis	Subcutaneous	Familial amyloid polyneuropathy	FDA approval in 2018
Volanesorsen (Waylivra)	ASO	Ionis	Subcutaneous	Familial chylomicronemia syndrome	EU approval in 2019
Patisiran (Onpattro)	siRNA	Alnylam	Intravenous	Polyneuropathy	FDA approval in 2018
Givosiran (Givlaari)	siRNA	Alnylam	Subcutaneous	Acute hepatic porphyria	FDA approval in 2019
Cobomarsen (MRG-106)	miRNA	miRagen (Viridian)	Intravenous/subcutaneous	Blood cancers	Phase II
Remlarsen (MRG-201)	miRNA	miRagen (Viridian)	Intradermal	Keloids	Phase II
MRG-110	miRNA	miRagen (Viridian)	Intradermal	Tissue Repair	Phase I
Pegaptanib (Macugen)	Aptamer (RNA)	Bausch + Lomb	Intravitreal	Macular Degeneration	FDA approval in 2014
Emapticap pegol (NOX-E36)	Aptamer (RNA)	NOXXON	Intravenous/Subcutaneous	Diabetic nephropathy, lung and pancreatic cancer	Phase II
Olaptesed pegol (NOX-A12)	Aptamer (RNA)	NOXXON	Intravenous	Brain cancer	Phase I/II
BNT162b2	mRNA	BioNTech and Pfizer	Intramuscular	COVID-19	FDA authorization for emergency use in 2020
mRNA-1273	mRNA	Moderna	Intramuscular	COVID-19	FDA authorization for emergency use in 2020
CVnCoV	mRNA	CureVac	Intramuscular	COVID-19	Phase III
AZD8601	mRNA	Moderna/Astrazeneca	Epicardial	Ischemic heart disease	Phase II
mRNA-1647	mRNA	Moderna	Intramuscular	Cytomegalovirus infection	Phase II
P-BCMA-101	mRNA	Poseida	Intravenous	Multiple myeloma	Phase II
mRNA-4157	mRNA	Moderna	Intramuscular	Cancer	Phase II
mRNA-3704	mRNA	Moderna	Intravenous	Methylmalonic aciduria	Phase I/II
MRT5005	mRNA	Translate Bio	Inhalation	Cystic Fibrosis	Phase I/II
mRNA-2416	mRNA	Moderna	Intratumoral	Solid tumors/lymphoma/advanced ovarian carcinoma	Phase I/II
BNT131 (SAR441000)	mRNA	BioNTech/Sanofi/Genmab	Intratumoral	Advanced melanoma	Phase I/II
Descartes-08	mRNA	Cartesian	Intravenous	Generalized myasthenia gravis	Phase I/II
BNT122	mRNA	BioNTech	Intravenous	Solid tumors	Phase I/II
mRNA-2752	mRNA	Moderna	Intratumoral	Solid tumors	Phase I
MEDI1191	mRNA	Moderna	Intratumoral	Solid tumors	Phase I
mRNA-1944	mRNA	Moderna	Intravenous	Chikungunya infection	Phase I
CV8102	mRNA	CureVac	Intratumoral	Solid tumors	Phase I
ARCT-810	mRNA	Arcturus	Intravenously	Urea disorder	Phase I
CV7202	mRNA	CureVac	Intramuscular	Rabies	Phase I
mRNA-1893	mRNA	Moderna	Intramuscular	Zika	Phase I
CV9202	mRNA	CureVac	Intradermal	Non-small cell lung cancer	Phase I
mRNA-5671	mRNA	Moderna	Intravenous	Cancer	Phase I
BNT111	mRNA	BioNTech	Intravenous	Advanced Melanoma	Phase I

*The table summarizes information available at ClinicalTrials.gov (https://clinicaltrials.gov/) as of January 26, 2021. ASO, antisense oligonucleotide; siRNA, small interfering RNA; miRNA, microRNA; RNA, ribonucleic acid; mRNA, messenger RNA.*

### Antisense Oligonucleotide (ASO), Small Interfering Rna (siRNA), and microRNA as Therapeutics

Antisense oligonucleotides are short single-stranded DNA, phosphorothioate DNA, RNA analogs, conformationally restricted nucleosides (locked nucleic acids, LNA), or morpholino phosphorodiamidate oligonucleotides complementary to a certain region of RNA that they are meant to target. The modifications in backbone, and sugar molecules give antisense oligos more affinity and stability (Bennett and Swayze, 2010). Zamecnick and Stephenson pioneered the field of antisense oligonucleotides in late 1970s. They designed and synthesized tridecamer deoxynucleotides complementary to reiterated terminal sequences of Rous sarcoma virus (RSV) at 3′ and 5′ ends. To test its function, RSV-infected chick embryo fibroblasts (CSF) were transfected with the tridecamer which hybridized to its complementary sequence in the target RNA (RSV RNA), thereby inhibiting viral production and cell transformation (Stephenson and Zamecnik, 1978).

There are two classes of ASOs: RNase H-dependent ASO (Wu et al., 2004), and RNase H-independent (steric block) ASO (Baker et al., 1997). The former is more commonly used and is dependent on the endogenous RNase H enzyme that hydrolyzes the RNA strand of an RNA/DNA duplex. The RNase H-dependent ASOs are more efficient in knockdown of gene expression than RNase H-independent ASOs (Larrouy et al., 1992; Dean et al., 1994; Dias and Stein, 2002). Minshull and Hunt (1986) were the first to demonstrate the RNase H-dependent ASO mechanism in reticulocyte lysate. They added RNase H-treated DNA-mRNA hybrid complexes directly to the reticulocyte lysate for translation, and observed a loss of full length translation products of the hybridized mRNA (Minshull and Hunt, 1986). Giles et al. (1995) further demonstrated the RNase H-mediated antisense effect in human leukemia cells by identifying the RNase H-generated mRNA fragments with a reverse ligation-mediated PCR method.

Steric block ASOs physically inhibit or prevent translation or splicing, and can be engineered to either prevent polyadenylation (Vickers et al., 2001), inhibit (Baker et al., 1997) or enhance translation (Liang et al., 2016), or alter splicing (Hua et al., 2007). For instance, Baker et al. (1997) designed RNase H-independent 2′-*O*-2-methoxyethyl ASOs that were complementary to the 5′ cap region of the intercellular adhesion molecule 1 (ICAM-1) transcript in human umbilical vein endothelial cells. These ASOs inhibited protein expression of the targeted transcript by interfering with the formation of the 80S translation initiation complex (Baker et al., 1997).

**FIGURE 2:**
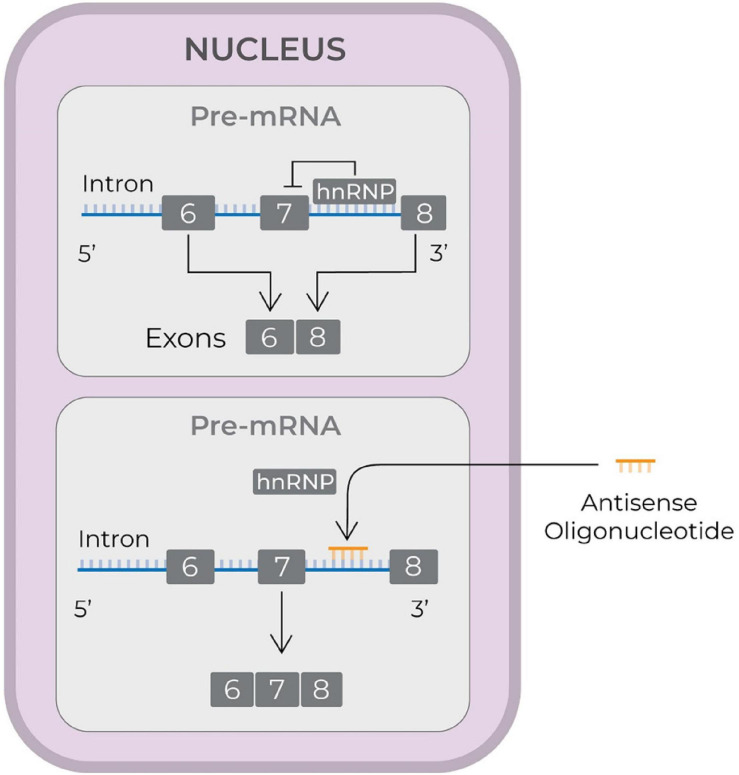
Schematic illustrating the mode of action of an antisense drug to treat spinal muscular atrophy. The antisense drugs reach nucleus, displace hnRNP proteins and increase the synthesis of transcripts containing exon 7 and thereby generate full length SMN protein (Rigo et al., 2012). SMN, survival of motor neuron; hnRNP, heterogeneous nuclear ribonucleoprotein; pre-mRNA, precursor mRNA; mRNA, messenger RNA; RNA, ribonucleic acid.

To date, three ASO drugs have received FDA approval: (1) nusinersen (Ionis Pharmaceuticals) (Neil and Bisaccia, 2019); (2) eteplirsen (Sarepta Therapeutics) (Lim et al., 2017); and (3) inotersen (Ionis Pharmaceuticals and Akcea Therapeutics) (Mathew and Wang, 2019). Nusinersen (FDA approval in December 2016) is used to treat spinal muscular atrophy (SMA), which is caused by deletions or mutations in the survival motor neuron 1 (SMN1) gene. The mutated or deleted gene does not produce enough SMN protein. SMN2, a homolog gene, is different from SMN1 only in that it is spliced with exon 8, rather than exon 7. This results in expression of small amount of full length SMN protein. Nusinersen is a 2′-*O*-methoxyethyl-phosphorothioate-modified drug that modulates splicing of SMN2 so as to increase the transcript containing exon 7 (see Figure 2, Rigo et al., 2012), and, therefore enhances production of full length SMN protein (Wurster and Ludolph, 2018).

Eteplirsen (Exondys 51, FDA approval in September 2016) is used to treat Duchenne muscular dystrophy (DMD). DMD is caused by mutations in the DMD gene that result in a premature stop codon and a non-functional dystrophin protein, with a mutation in exon 51 being most frequent single exon mutation (Bladen et al., 2015). Eteplirsen is 30-mer phosphomorpholidate oligonucleotide that recognizes and hybridizes to exon 51 of the DMD gene thereby modulating the pre-messenger RNA splicing process to exclude exon 51 from the mature mRNA. This restores the reading frame of the DMD gene and a shortened but functional dystrophin protein is produced (Lim et al., 2017).

Inotersen (Tegsedi, FDA approval in October 2018) is used to treat familial amyloid polyneuropathy caused by the mutation in the transthyretin (TTR) gene. The mutated gene leads to translation and accumulation of the abnormal TTR protein in different organs, including heart, kidney, eyes, and nerves. Tegsedi is a 20-mer chemically modified RNA molecule that hybridizes to TTR mRNA, mimicking a DNA/RNA complex, and thereby interferes with TTR protein production (Mathew and Wang, 2019).

Volanesorsen (Ionis Pharmaceuticals) was recently approved in Europe (Waylivra, EU conditional marketing authorization in May 2019). This is an antisense oligonucleotide indicated for treatment of familial chylomicronemia syndrome (Paik and Duggan, 2019). It recognizes and hybridizes to the complementary sequence in the 3′ untranslated region of apolipoprotein CIII mRNA to increase its degradation by RNase H1.

**FIGURE 3:**
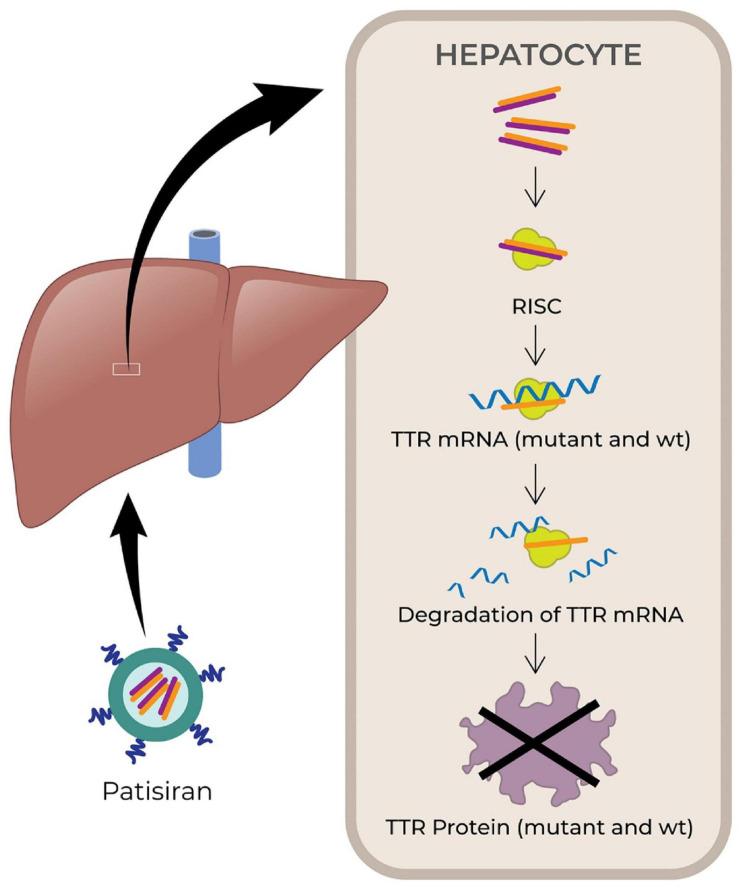
Schematic illustrating the mechanism of action of small interfering RNA (siRNA) drug, patisiran. The drugs are encapsulated in lipid nanoparticles and administered intravenously. After administration, the drugs finally reach hepatocyte and released into the cytoplasm, where it is loaded onto the RISC. The antisense strand hybridizes with target mRNA to suppress the production of target protein (TTR) (Kristen et al., 2018). TTR, transthyretin; wt, wild type; RISC, RNA-induced silencing complex; mRNA, messenger RNA; RNA, ribonucleic acid.

Small interfering RNAs (siRNAs) are small non-coding RNA duplexes that originate from precursor siRNAs. The latter are either transcribed or artificially introduced and range from 30 bp to more than 100 bp. The precursor siRNA duplex is processed by the endogenous Dicer enzyme into 20–30 bp long siRNA with two base overhangs in the 3′ region, which interacts with the endogenous RNA-induced silencing complex (RISC) to elicit RNA interference (RNAi). The endonuclease argonaute 2 (AGO2) component of the RISC cleaves the sense strand, leaving intact the antisense strand, which guides the active RISC to its target mRNA. Then AGO2 cleaves the phosphodiester backbone of the target mRNA. The antisense strand is usually fully complementary to the coding region of the target mRNA, therefore siRNA knocks down one specific target gene (Wittrup and Lieberman, 2015; Dana et al., 2017). Fire et al. (1998) were first to demonstrate RNAi by blocking the expression of unc-22, unc-54, fem1, and hlh-1 genes in *C. elegans*. They showed that dsRNA are more effective than ssRNA to artificially induce RNAi and destroy an mRNA target (Fire et al., 1998; Lam et al., 2015). Patisiran (Onpattro, Alnylam Pharmaceuticals, FDA approval in August 2018) was the first marketed siRNA-based drug (see Figure 3, Kristen et al., 2018). It is used to treat adult patients with polyneuropathy caused by hereditary TTR-mediated amyloidosis. Patisiran is a dsRNA that acts through RNAi and induces degradation of mRNA encoding TTR (Kristen et al., 2018). Recently, another siRNA drug, Givosiran (Givlaari, Alnylam Pharmaceuticals) received FDA approval in November 2019 (Alnylam, 2020b) for the treatment of acute hepatic porphyria. The drug targets aminolevulinate synthase 1 mRNA in liver, and reduces the levels of disease-causing neurotoxic intermediates aminolevulinic acid and porphobilinogen (Alnylam, 2020b; Givlaari, 2020).

Unlike siRNAs, microRNAs (miRNAs) are small non-coding RNA molecules that regulate the expression of multiple mRNAs by blocking translation or promoting degradation of the target mRNAs. They were first discovered in *C. elegans* (Lee et al., 1993). This class of non-coding RNAs are transcribed from genomic DNA as primary miRNAs (pri-miRNAs). The latter adopt a loop structure with interspersed mismatches and are cleaved by Drosha to a 70–100 bp precursor miRNAs (pre-miRNAs), before leaving the nucleus. Exportin 5 transports the pre-miRNAs to the cytoplasm, where Dicer processes them into 18–25 bp RNA duplexes with two base overhangs in the 3′ region. These structures are now referred to as miRNAs. The miRNA is then loaded into the RISC to form a miRISC complex. The miRNA duplex unwinds to release the sense strand. The antisense strand guides the miRISC. Hybridization usually occurs at 2–7 bases of the 5′ end of miRNA and the 3′ UTR of the target mRNA. The target mRNA is inhibited via translational repression, degradation or cleavage (Lam et al., 2015; Lou et al., 2018; O’Brien et al., 2018). The miRNA-based therapeutics could be categorized into two types: miRNAs mimics and miRNAs inhibitors. The former are double-stranded RNA molecules that mimic miRNAs, while the latter are single-stranded RNA oligos designed to interfere with miRNAs. Although there are no miRNA-based drugs on the market as of today, some promising candidates are currently in clinical trials. For instance, miRagen Therapeutics (Viridian Therapeutics) is developing cobomarsen (MRG-106), remlarsen (MRG-201), MRG-229 (miR-29 mimic; pre-clinical), and MRG-110 (miR-92 inhibitor)(Pipeline – miRagen, 2019; Pipeline – Viridian, 2021). Cobomarsen (MRG-106) is designed to treat patients with blood cancers by reducing miR-155 levels (Pipeline – miRagen, 2019; Pipeline – Viridian, 2021). Remlarsen (MRG-201) is designed to mimic miR-29 to treat keloids (ClinicalTrials.gov, 2019; Hanna et al., 2019; Pipeline – miRagen, 2019; Pipeline – Viridian, 2021), MRG-229 mimics miR-29 to treat pathologic fibrosis (Hanna et al., 2019; Pipeline – miRagen, 2019; Pipeline – Viridian, 2021), while MRG-110 inhibits miR-92 to accelerate tissue repair (Hanna et al., 2019; Pipeline – miRagen, 2019; Pipeline – Viridian, 2021). InteRNA Technologies has designed INT-1B3 to mimic tumor suppressor miRNA in order to modulate the immunosuppressive tumor microenvironment and treat solid cancers (Yahyanejad et al., 2018; Businesswire, 2019). Regulus Therapeutics’ product RG-012 inhibits miR-21 to treat Alport Syndrome (Regulus, 2019). Besides RG-012, Regulus has several other miRNA-based drugs in the pipeline to treat ADPKD (miR-17), glioblastoma multiforme (miR-10b), and others (Regulus, 2019). Miravirsen, which is under development by Roche Innovation Center Copenhagen, targets miR-122 and decreases viral load in patients with chronic hepatitis C (van der Ree et al., 2016). Another candidate, a TargomiR drug MesomiR-1 (EnGeneIC Limited) mimics tumor suppressor miR-16 to treat patients with mesothelioma (Reid et al., 2016). Overall, both siRNAs and miRNAs are attractive candidates as therapeutic agents and may overcome the major limitations of small molecule-based and protein-based drugs.

### Aptamers as Therapeutics

Aptamers are short single-stranded nucleic acids that can bind to variety of targets, such as proteins, peptides, carbohydrates, and other molecules, by virtue of the tertiary structure of the aptamer, rather than its sequence. Aptamers were first described in 1990 independently by two groups, Ellington and Szostak (1990) and Tuerk and Gold (1990), through systematic evolution of ligand by exponential enrichment (SELEX) technique. Aptamers are evolved from highly diverse nucleic acid pools to bind to the targets with high specificity and affinity, and can serve as agonists (Dollins et al., 2008; McNamara et al., 2008), antagonists (Santulli-Marotto et al., 2003; Berezhnoy et al., 2012), bispecific aptamers (Schrand et al., 2014, 2017), and even carriers for other drugs (McNamara et al., 2006; Lozano et al., 2016).

**FIGURE 4:**
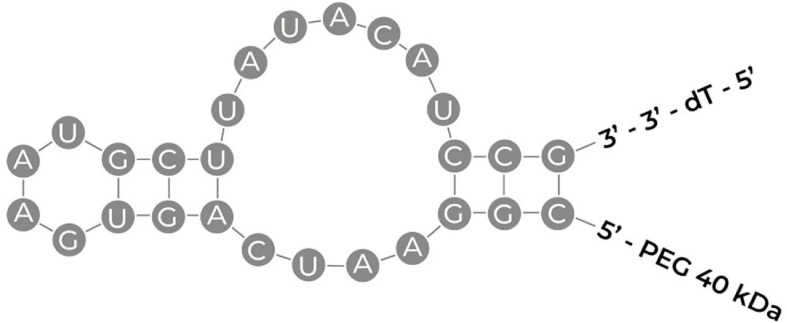
Schematic illustrating the sequence of pegaptanib with its secondary structure. PEG, polyethylene glycol; dT, deoxthymidine (Ng et al., 2006; Amadio et al., 2016).

Although aptamers hold promise as therapeutics, there is only one FDA-approved aptamer-based drug on market. Pegaptanib (Macugen, Bausch + Lomb Pharmaceutical Retina Portfolio, FDA approval in December 2004) is a 27 base RNA aptamer (see Figure 4 for predicted structure, Ng et al., 2006) selected against vascular endothelial growth factor (VEGF) and is used to treat age-related macular degeneration, a leading cause of irreversible blindness worldwide (Ruckman et al., 1998). Many other aptamer-based drugs are currently in clinical trials and have been recently reviewed elsewhere (Kaur et al., 2018). Emapticap pegol (NOXXON, 2020b; NOXXON Pharma), for instance, was evolved to specifically bind and inhibit the pro-inflammatory chemokine C-C motif-ligand 2 (CCL2) to treat diabetic nephropathy as well as lung and pancreatic cancer (NOXXON, 2020b). Similarly, Olaptesed pegol (NOXXON, 2020a; NOXXON Pharma) was evolved to target CXC chemokine ligand (CXCL12) for clinical treatment of brain cancer (glioblastoma/glioma). REG1 is an anticoagulation combination therapy that consists of pegnivacogin, an RNA aptamer inhibitor of coagulation factor IXa, and anivamersen, a complementary sequence to rapidly reverse the anticoagulant effect of pegnivacogin (Lincoff et al., 2016). The intent of this combination aptamer is that the first will induce anticoagulation as needed, whereas the second will reverse the anticoagulation as necessary to avoid excessive bleeding. However, in a trial comparing the aptamer therapy against the approved anticoagulant bivalirudin, in patients undergoing percutaneous coronary artery intervention, REG1 did not provide any greater benefit, and had a greater frequency of serious allergic reactions. However, the concept of having a second aptamer to act as an antidote to the first, so as to treat side effects or adverse reactions is an intriguing idea.

Aptamers have great potential to replace monoclonal antibodies in therapeutic and diagnostic (Damase et al., 2018; Bauer et al., 2019) applications because they can be produced via chemical synthesis, are more cost-effective in manufacturing, simpler to modify, and elicit little immunogenicity. However, despite the fact that aptamers have many advantages over antibodies, their clinical translation is challenging due to suboptimal pharmacokinetic properties (highly sensitive to nucleases, readily excreted by kidneys) and complexity of selection techniques (a time consuming process with low success rates) (Nimjee et al., 2017).

### mRNA as Therapeutics

Messenger RNAs (mRNAs) serve as the intermediates between the coding genomic DNA and the encoded proteins (Gilbert, 1986). Essentially, mRNAs are transient blueprints of genes that are encoded in the genomic DNA. The mRNA transcripts of these genes deliver the genetic information to the translational machinery to generate the encoded proteins (Wolff et al., 1990; Conry et al., 1995). Wolff et al. (1990) were amongst the first to use exogenous mRNA to induce the expression of a protein *in vivo*. They injected synthetic mRNA encoding luciferase, chloramphenicol acetyltransferase, or beta-galactosidase into mouse skeletal muscle and detected proteins translated from these RNAs at the injection site. Importantly, the extent of expression from RNA was comparable to that obtained from injection of a DNA vector encoding the same proteins (Wolff et al., 1990). Jirikowski et al. (1992) demonstrated the therapeutic effect of mRNA in a rodent model of central diabetes insipidus. Brattleboro rats have a genetic deficiency of vasopressin. The investigators purified cytoplasmic mRNA from wild-type rat hypothalamus, or synthesized mRNA that encodes for vasopressin. Injection of either mRNA into the hypothalamus of Brattleboro rats induced the synthesis of vasopressin and transiently reversed diabetes insipidus (Jirikowski et al., 1992).

**FIGURE 5:**
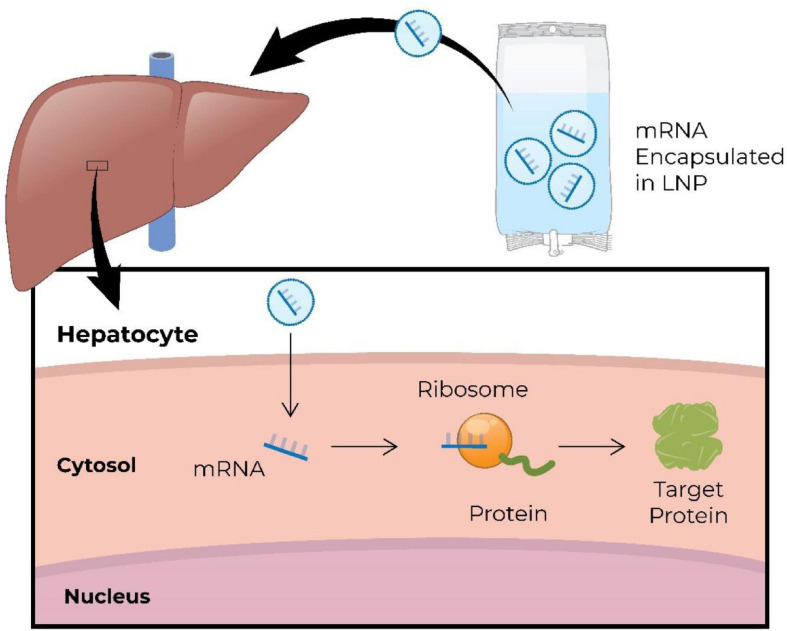
Schematic illustration of intravenous administration of mRNA encapsulated in lipid nanoparticles (LNP) to restore missing/defective protein in hepatic cells (An et al., 2017). mRNA, messenger RNA; RNA, ribonucleic acid.

Synthetic or *in vitro*-translated mRNA is engineered to mimic naturally occurring mRNA (Jirikowski et al., 1992; Conry et al., 1995). It consists of a single-stranded open reading frame flanked by untranslated regions, and has a 5′ cap for translation, and a 3′ poly(A) tail for stability (Conry et al., 1995; Whisenand et al., 2017; Shin et al., 2018). Exogenous mRNA is translated into protein in the cytoplasm, and degrades within the cytoplasm typically within minutes to hours, posing no risk of integration into the genome. Figure 5 demonstrates systemically delivered mRNA expressing hepatic protein (An et al., 2017). Development and manufacturing of RNA therapeutics is relatively simple and a more cost-effective process comparing to that for recombinant proteins or small molecules. In addition, RNA sequences can be easily modified allowing for personalization of RNA therapy. There are several therapeutic modalities that utilize mRNA: (1) replacement therapy, where mRNA is administered to the patient to compensate for a defective gene/protein, or to supply therapeutic proteins; (2) vaccination, where mRNA encoding specific antigen(s) is administered to elicit protective immunity; (3) cell therapy, where mRNA is transfected into the cells *ex vivo* to alter cell phenotype or function, and then these cells are delivered into the patient.

#### mRNA as Replacement Therapy

Currently, there are three major biopharmaceutical companies that develop mRNA therapeutics: Moderna (Boston, MA, United States), CureVac (Tubingen, Germany), and BioNTech (Mainz, Germany). Together, they have a diverse portfolio of gene therapy products in the pipeline, covering metabolic diseases, heart diseases, and immunomodulators for applications in immuno-oncology. For instance, Moderna’s AZD8601 is a naked mRNA encoding vascular endothelial growth factor (VEGF-A) that is intended to be delivered through epicardial injection during coronary artery bypass surgery. The intent is to enhance local angiogenesis, thereby reducing myocardial ischemia and improving left ventricular systolic function (Carlsson et al., 2018) in patients with ischemic heart disease. The efficacy of this drug is being evaluated in a phase II trial conducted by AstraZeneca. Another therapeutic candidate, mRNA-3704 (Moderna) encodes methylmalonyl-CoA mutase and is designed to treat the deficiency of this enzyme that leads to methylmalonic aciduria (Modernatx, 2021a). MRT5005 (Translate Bio) is being developed to treat cystic fibrosis, an inherited disease caused by a mutation in the cystic fibrosis transmembrane conductance regulator (CFTR), a chloride channel. When it is disrupted in the epithelium, there is an accumulation of thickened mucous in different organs, including the pancreas and lungs. MRT5005 encodes fully functional CFTR and is delivered to lung epithelial cells through nebulization (TranslateBio, 2019). Other therapeutics being developed by Moderna include mRNA-2416, mRNA-2752, and MEDI1191, which are mRNA-based immunomodulators. The mRNA-2416 encodes OX40 Ligand (OX40L), which is a co-stimulatory membrane-bound protein that enhances the expansion, function and survival of T cells to mount an attack against cancer cells. When delivered by intratumoral injection (solid tumors/lymphoma/advanced ovarian carcinoma), cells in the tumor may express this ligand on their surfaces, leading to a stronger T cell attack against the tumor cells. Additionally, Moderna is investigating whether mRNA-2416 has the potential to elicit an abscopal effect in metastatic cancer, in which localized injection into a tumor, and cytolytic release of tumor antigens, would generate a secondary immune response and have an effect on metastases throughout the body (Moderna, 2020). Another therapeutic candidate, mRNA-2752, delivers OX40L into the tumors, as well as mRNAs encoding immunostimulatory cytokines IL-23 and IL-36γ to further promote T cell-mediated cytotoxicity. The mRNA drug MEDI1191 is indicated for the solid tumors as well, and encodes IL-12, one of the most potent cytokines in mediating antitumor activity (Tugues et al., 2015).

BioNTech, in collaboration with Sanofi, is evaluating an mRNA-based intratumoral immunomodulator BNT131 (SAR441000) as a monotherapy in patients with advanced melanoma. This immunomodulator is a combination of IL-12sc, IL-15sushi, IFNα, and GM-CSF mRNAs, increased concentration of which in the local tumor microenvironment may promote natural killer cell activation and induce cytotoxic T-cell responses, resulting in an immune-mediated destruction of tumor cells (National Cancer Institute, 2011; Pipeline – BioNTech, 2021). The mRNA drug CV8102 from CureVac is a TLR7/8/RIG1 agonist designed to induce a systemic immune response to the injected primary tumor as well as distant metastases of some solid tumors. This agent is targeted against advanced melanoma, cutaneous squamous cell carcinoma, and squamous cell carcinoma of head and neck (Pipeline – CureVac, 2020).

Although the aforementioned pharmaceutical companies were pioneers in commercialization of mRNA therapeutics, there are many other research groups and biotech start-ups worldwide entering the field. For instance, Connolly et al. (2018) have proposed mRNA-based therapy for α-1-antitrypsin deficiency. This genetic disorder is characterized by destruction of lung tissue by neutrophil elastase due to lack of its natural inhibitor α-1-antitrypsin and by liver damage due to deposition of defective α-1-antitrypsin. The latter is normally produced by the liver to be transported to the lungs, and is encoded by the SERPINA1 gene, a mutation in which leads to defects in transport and activity. Human SERPINA1-encoding mRNA systemically delivered to wild-type mice resulted in target protein expression in both liver and lungs (Connolly et al., 2018).

Cao et al. (2019) recently developed an mRNA drug for citrin deficiency. This disorder is caused by a mutation in the SLC25A13 gene, which encodes citrin, a mitochondrial membrane transport protein involved in the urea cycle. Citrin deficiency leads to hyperammonemia and neuropsychiatric disturbances. Intravenous administration of a human citrin-encoding mRNA in SLC25A13-knockout mice reduced hepatic citrulline and blood ammonia levels following an oral sucrose challenge, and reduced sucrose aversion, a hallmark of citrin deficiency (Cao et al., 2019). Another mRNA drug for a disorder of the urea cycle is being developed by Arcturus Therapeutics. Their mRNA-based therapeutic ARCT-810 encodes ornithine transcarbamylase (OTC) to treat the genetic deficiency of this enzyme and is now in early clinical trials (ClinicalTrials.gov, 2020b). Zhu et al. (2019) reported encouraging data from preclinical studies of an mRNA therapy for Fabry disease. The latter is caused by mutations in the GLA gene that encodes enzyme α-galactosidase, required for utilization of glycolipids. In Fabry disease, glycolipid derivates (globotriaosylceramide, globotriaosylsphingosine) accumulate over time in multiple tissues leading to range of clinical symptoms. A single dose of GLA mRNA intravenously administered in GLA-deficient mice significantly reduced accumulation of globotriaosylsphingosine in plasma and tissues. Of note, this beneficial effect of mRNA was observed for up to 6 weeks after administration (Zhu et al., 2019).

#### mRNA Vaccines

mRNAs encoding antigens and/or adjuvants can be used as vaccines to evoke protective immunity against infectious diseases (prophylactic vaccines), or to harness the immune system to fight cancer (therapeutic vaccines). The COVID-19 pandemic has showcased the utility and advantages of RNA technology for vaccination, as out of all COVID-19 vaccines under development, the first two to have received emergency use authorization by the FDA were RNA-based. The first authorized vaccine was developed by BioNTech in collaboration with Pfizer. Initial clinical trials conducted in Germany helped to identify two leading vaccine candidates, BNT162b1 and BNT162b2, which were then tested further in the US (Walsh et al., 2020a, b). BNT162b1 encodes secreted trimerized receptor binding domain of spike protein, whereas BNT162b2 encodes membrane-anchored full length spike protein modified by two proline mutations to stabilize the prefusion conformation. Both RNA candidates incorporate 1-methyl-pseudouridine, which dampens innate immune sensing and increases mRNA translation *in vivo* (Karikó et al., 2008) and are formulated in the lipid nanoparticles (refer to Delivery of RNA therapeutics section below for details). The safety and immunogenicity data of these two vaccine candidates in younger and older adults supported advancement of BNT162b2 into subsequent trials (Mulligan et al., 2020). A large Phase III clinical trial revealed extraordinarily high efficacy (with vaccination preventing about 90% of symptomatic infections) with excellent safety, leading to emergency use authorization by the FDA on December 11, 2020 (FDA, 2021b).

The Moderna RNA vaccine against COVID-19 received emergency use authorization shortly thereafter (December 18, 2020) (FDA, 2021a), based on a large Phase III trial showing at least equal efficacy and safety as BNT162b2. Similarly to BNT162b2, this vaccine encodes stabilized SARS-CoV-2 spike immunogen, and like the BNT mRNA, is delivered intramuscularly as a lipid nanoparticle-encapsulated mRNA (Corbett et al., 2020; Jackson et al., 2020). The development timeline of both vaccines was unprecedented. It was reported that it took merely 2 days after the SARS-CoV-2 genome sequence by Chinese scientists in January 2020 (Novel 2019 Coronavirus Genome, 2020; Wang et al., 2020, p. 2), to select the appropriate sequence for the Moderna vaccine candidate, 25 days to manufacture the first clinical batch of mRNA-1273, and another 35 days to dose the first participant (Jackson et al., 2020; Modernatx, 2021b). Other biotech companies are developing mRNA-based vaccines to address the COVID-19 pandemic. For instance, at the time of this writing, CureVac has initiated a Phase 3 clinical trial of its SARS-CoV-2 vaccine candidate CVnCoV (Pipeline – CureVac, 2020).

The biotechnology response to the COVID-19 pandemic has highlighted the speed and flexibility of mRNA vaccines (Vogel and Sarver, 1995), and reveals mRNA therapeutics to be a powerful tool to address epidemic outbreaks caused by newly emerging viruses. The relative simplicity of the development process and flexibility of the manufacturing platform can markedly accelerate clinical development (Liu, 2019). As such, mRNA-based vaccine technology has attracted a lot of attention during the COVID-19 pandemic (Le et al., 2020; Lurie et al., 2020). Unlike viral vector-based vaccines, mRNA vaccines are not confounded by pre-existing immunity against the vector (Ura et al., 2014; Condit et al., 2016). They also do not need to enter the nucleus for translation, unlike plasmid DNA vaccines that require nuclear localization to elicit their protective effects (Ulmer et al., 1993; Ledwith et al., 2000).

RNA vaccines can activate both cell-mediated and humoral immunity. For instance, Martinon et al. (1993) demonstrated induction of anti-influenza cytotoxic T lymphocytes *in vivo* by immunizing animals with subcutaneous injection of liposome-encapsulated mRNA, which encoded the nucleoprotein of the influenza virus. Petsch et al. (2012) evaluated effects of a multi-valent mRNA vaccine (hemagglutinin, neuraminidase, and nucleoprotein) against influenza A H1N1, H3N2, and H5N1 strains in mice, ferrets, and pigs. This intradermally injected mRNA vaccine induced antigen-specific neutralizing antibodies and protected the animals from influenza A virus (Petsch et al., 2012). Similarly, the intramuscularly administered vaccines against avian influenza A viruses H10N8 and H7N9 have been shown to be safe and immunogenic in first-in-man trials conducted by Moderna (Feldman et al., 2019). Moderna is currently evaluating vaccine candidates for other infectious respiratory diseases: respiratory syncytial virus (mRNA-1172, mRNA-1777) and metapneumovirus/parainfluenza 3 (mRNA-1653).

The protective effects of mRNA vaccines have been also proven useful beyond respiratory pathogens. For example, Schnee et al. (2016) demonstrated the potency of mRNA vaccines against rabies in rodents and pigs. This vaccine against a viral glycoprotein induced antigen-specific immune responses *in vivo* (Schnee et al., 2016). Notably, specific CD4+ T cells induced by the mRNA vaccine were higher than those induced by a licensed vaccine, and the titers of neutralizing antibody in mice remained stable for the entire observation period (up to 1 year). The safety, reactogenicity and immune response of this vaccine is currently being evaluated in a phase I trial conducted by CureVac (CV7202) (ClinicalTrials.gov, 2020c). Pardi et al. (2017) proposed a bi-valent modified mRNA vaccine that encodes pre-membrane and envelope glycoproteins of a Zika virus strain responsible for the outbreak in 2013. A single dose of this vaccine, encapsulated in lipid nanoparticles and delivered intradermally, was sufficient to protect mice against viral challenges at 2 weeks or 5 months after vaccination, and non-human primates at 5 weeks after vaccination (Pardi et al., 2017). Utilizing the same antigens, Moderna has been developing a non-modified encapsulated mRNA-1893 vaccine against Zika, which received fast track FDA designation and is currently in phase I trials (ClinicalTrials.gov, 2020f) to assess its safety, tolerability and immunogenicity. Importantly, mRNA-1893 had prevented congenital transmission of the virus in mouse models of congenital infection (Jagger et al., 2019). John et al. designed a cytomegalovirus (CMV) vaccine to prevent CMV infection and/or disease during pregnancy and in transplant patients. The vaccine consists of six modified mRNA constructs encoding CMV glycoproteins and pentameric complex and is delivered intramuscularly in lipid nanoparticles. Its single dose elicited robust immune response in mice and non-human primates (John et al., 2018), and is currently in clinical trials (mRNA-1647) sponsored by Moderna (ModernaTX Inc, 2019a).

In addition to mounting active immunity, mRNA can be also used for passive immunization. A drug candidate mRNA-1944 (Moderna) is a great example of mRNA therapeutics encoding human monoclonal neutralizing antibodies (mAb). This drug is designed to provide passive protection against chikungunya infection. The ultrapotent antibodies were isolated from the B cells of a survivor of natural infection, and their sequences were encoded into mRNA molecules, encapsulated in lipid nanoparticles and delivered by infusion into mice. After mRNA delivery, one human mAb, CHKV-24, was found to be expressed at immunologically relevant levels, and its protective capacity was evaluated in mouse models of chikungunya. Treatment with CHKV-24 mRNA reduced viremia to undetectable levels at 2 days after inoculation and protected mice from mortality. Further studies with non-human primates also demonstrated a long-lasting immunogenic effects of the drug (Kose et al., 2019). Overall, the pre-clinical data encouraged a first-in-man trial (ModernaTX Inc, 2019b).

**FIGURE 6:**
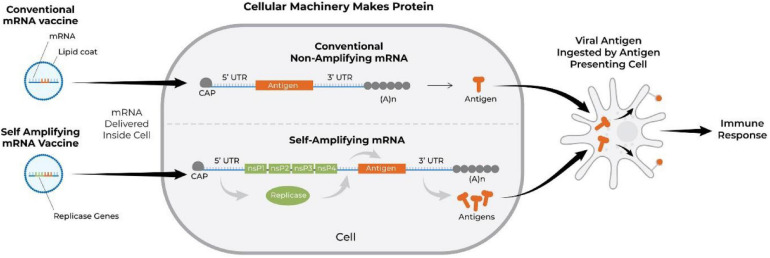
Schematic showing the mechanism of action of conventional (BioNTech and Moderna COVID-19 vaccines) and self-amplifying mRNA vaccines. The mRNA vaccine is translated into protein, processed by antigen presenting cells, and subsequently activates immune responses. nsP, non-structural protein; Cap, *N7*-methylated guanosine structure covalently joined to the first nucleotide of the mRNA through a reverse 5′ to 5′ triphosphate linkage (Kowalski et al., 2019; Dolgin, 2021); A, adenosine molecule; (A)n, poly-A tail; UTR, untranslated region.

Another interesting RNA-based approach to vaccination is that with self-amplifying RNA vaccines. The backbone sequence for the latter is adapted from an alphavirus, a positive-sense single-stranded RNA virus with high capacity for replication (Lundstrom, 2018b; Vogel et al., 2018). Such mRNA vaccine contains an antigen-encoding sequence and viral RNA dependent RNA polymerase-encoding sequence along with other elements required for replication (Hyde et al., 2015). The advantage of the self-replicating approach is that significantly higher amount of antigen can be expressed with lower doses of mRNA (Vogel et al., 2018). Both types of RNA vaccines degrade after transient expression; however, the self-replicating RNA achieves longer term expression of the antigen (Ulmer and Geall, 2016). Figure 6 demonstrates the mode of action of conventional and self-amplifying mRNA vaccines (Kowalski et al., 2019). Harvey et al. (2003) were among the first to demonstrate the ability of self-amplifying mRNA vaccines to elicit antigen-specific immunity. They constructed vaccine vectors based on replicon RNA of the Australian flavivirus Kunjin that encoded HIV-1 Gag antigen and delivered them intramuscularly as either naked RNA or in the form of virus-like particles in BALB/c mice. Such immunization induced both Gag-specific antibody and protective Gag-specific CD8+ T-cell responses (Anraku et al., 2002; Harvey et al., 2003). Similarly, Moyo et al. (2018) engineered a vaccine against HIV-1 using an RNA replicon derived from Semliki Forest virus, which encoded 6 highly conserved regions of HIV-1 proteins. The self-replication approach was also used by McKay et al. (2020) (Imperial College London) to develop another mRNA COVID-19 vaccine which encodes the pre-fusion stabilized SARS-CoV-2 spike protein, which vaccine has now entered a phase I trial.

There are multiple RNA cancer vaccines in clinical trials such as CV9202 (CureVac), which is a self-adjuvating RNA vaccine targeting six antigens commonly expressed in non-small cell lung cancer (Sebastian et al., 2014). The mRNA-5671 (Moderna) targets KRAS and is being currently evaluated in patients with KRAS-mutant advanced or metastatic non-small cell lung cancer, colorectal cancer, or pancreatic adenocarcinoma (ClinicalTrials.gov, 2020e). The mRNA-4157 is another cancer vaccine from Moderna, but, unlike mRNA-5671, it is an individualized therapeutic vaccine against melanoma. In this approach, genetic sequencing and bioinformatic analysis is performed on a patient’s tumor to identify 20 patient-specific neoantigen epitopes which are encoded by an mRNA construct manufactured for a single patient (National Cancer Institute, 2011; ClinicalTrials.gov, 2020a; van Dülmen and Rentmeister, 2020). Another example of the individualized cancer vaccine in clinical trials is BNT122 (phase II) from BioNTech, which is designed for locally advanced or metastatic solid tumors (including melanoma, non-small cell lung cancer, bladder cancer, and others). BioNTech has many other anti-cancer vaccines in their pipeline, with the following ones having reached clinical studies: BNT111 for advanced melanoma; BNT112 for metastatic castration-resistant prostate cancer and high-risk localized prostate cancer; BNT113 against HPV16-derived oncoproteins E6 and E7 found in HPV16-positive solid cancers, such as head and neck squamous cell carcinoma; BNT114 for triple negative breast cancer; and BNT115 for ovarian cancer (Pipeline – BioNTech, 2021).

#### mRNA-Enhanced Cell Therapies

**FIGURE 7:**
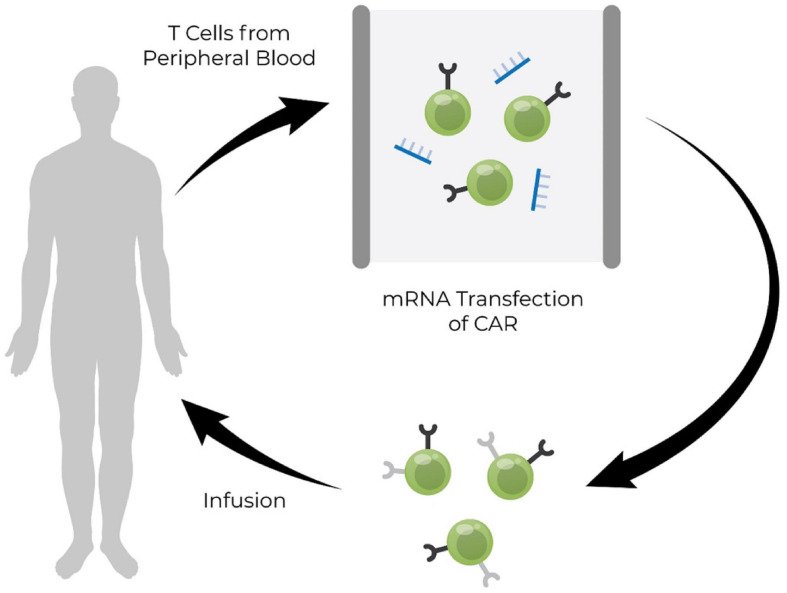
Schematic illustrations of the use of mRNA in engineering cells *ex vivo*. T cells derived from peripheral blood of patients suffering from disease are modified *ex vivo* with mRNA expressing the chimeric antigen receptor (CAR) and then modified cells are reinfused into the patient (Bertoletti and Tan, 2020). mRNA, messenger RNA; RNA, ribonucleic acid.

Cell therapy may be enhanced by mRNA. The cells may be obtained from the patient or the cell bank. Subsequently, they are therapeutically modified *ex vivo* with mRNA encoding the desired proteins (such as CARs, reprogramming or transdifferentiation factors, telomerase, etc.). Then the mRNA-enhanced cells are re-infused into the patient to treat the disease (see Figure 7, Bertoletti and Tan, 2020). Currently, there are several cell-based therapies that have reached clinical trials and employ mRNA. For example, TriMix-based immunotherapy (ECI-006) is a combination of mRNAs encoding DC-activating molecules (CD40L, CD70, and caTLR4) and melanoma-specific tumor-associated antigens (tyrosinase, gp100, MAGE-A3, MAGE-C2, and PRAME) (Arance Fernandez et al., 2019, p. 011) that are transfected into autologous DCs *ex vivo*. This therapy has demonstrated significant clinical activity in combination with ipilimumab without increasing regimen toxicity in metastatic melanoma (De Keersmaecker et al., 2020). MCY-M11 (MaxCyte) is an autologous CAR-T cell therapy for mesothelin-expressing solid tumors. The peripheral blood lymphocytes from a single leukapheresis are transfected with anti-human mesothelin mRNA CAR and cryopreserved as multiple cell aliquots for repeat administrations (Hung et al., 2018). Descartes (Cartesian Therapeutics) is an autologous cell therapy, where anti-BCMA CAR T-cells are engineered *ex vivo* by transient expression of mRNA, to treat myasthenia gravis and relapsed/refractory multiple myeloma (SparkCures, 2020). Although mRNA in aforementioned therapies is used to achieve transient expression of proteins, it can also be designed to serve as a gene editing tool to achieve stable expression of target proteins.

It is also possible to combine mRNA and DNA into a therapeutic. In one approach, a transposon system is used to genetically modify cells for therapy. This system consists of a DNA plasmid which encodes a gene of interest flanked by a mirrored set of inverted repeats (transposon), together with mRNA encoding the transposase enzyme. The plasmid DNA encoding the transposon is co-delivered along with the mRNA transposase enzyme in a single electroporation reaction. The transposase that is translated from the mRNA then binds to the inverted repeats and cuts the DNA, to release the transposon. After that, the transposon binds a strand of genomic DNA with a TA dinucleotide, where the transposase creates a double-stranded break, allowing the transposon to integrate (Singh et al., 2014). Using this approach, Poseida Therapeutics has developed a cell therapy for multiple myeloma, P-BCMA-101, which is now in clinical trials. The company’s proprietary mRNA-based transposon system is used to integrate a transposon encoding the anti-BCMA CAR into resting T cells (Singh et al., 2014, p. 19; Ostertag, 2018).

## Delivery of RNA Therapeutics

Targeted delivery is a major hurdle for effective RNA Therapeutics, a hurdle that must be overcome to broaden the application of clinical translation of this type of therapeutic. Because mRNA is inherently unstable it requires delivery vehicles that will protect the cargo from RNAase degradation. There is a need for novel delivery vehicles that will deliver the RNA drug to the site of therapeutic action facilitating the entry of the RNA drug into the cytoplasm where it may exert its effect. In the following paragraphs we provide a brief review of the advances made in RNA delivery vehicles.

### Lipid-Based Nanoparticles

**FIGURE 8:**
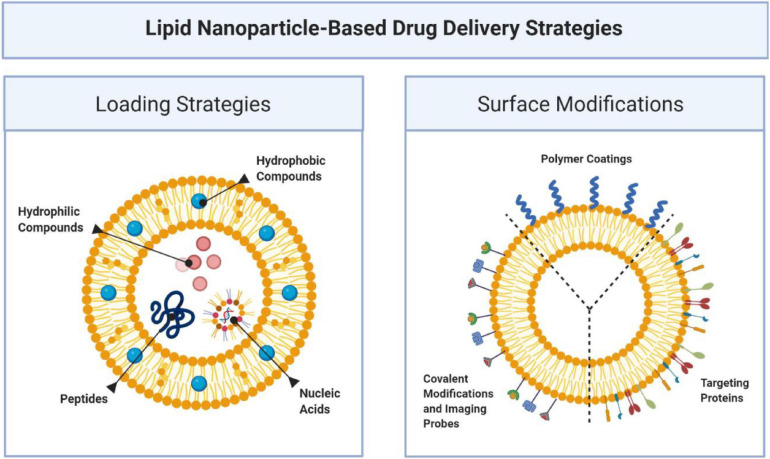
Lipid based nanoparticles accommodate a variety of therapeutic payloads including hydrophobic and hydrophilic small molecules, genetic materials, and proteins. One of the advantages of this platform is its ability to functionalize the surface with various proteins that can serve to target and localize nanoparticles over specific targets, imaging probes or covalent modification (Sushnitha et al., 2020; Zinger et al., 2020).

Liposomes are formed when materials containing polar head groups and non-polar tails (phospholipids) are dispersed in aqueous phase. They are spherical vesicles consisting of at least one phospholipid bilayer enclosing an aqueous core. They are a flexible drug delivery particle which may have various surface modifications (see Figure 8, Sushnitha et al., 2020; Zinger et al., 2020) capable of delivering a variety of therapeutic payloads (Torchilin, 2005). Most notably amongst these application is Doxil [Barenholz (Chezy), 2012] a liposome encapsulated formulation of doxorubicin for the treatment of cancer, that reduces heart toxicity related to the drug. This breakthrough treatment demonstrated the efficacy of the use of nanoparticles to change the biodistribution of drug and increase its safety.

Liposomes have an inherent advantage in that they mimic cell membrane composition and can encapsulate mRNA when combined with cationic lipids. Positively charged lipids can electrostatically interact with negatively charged mRNA to form complexes of RNA and liposomes. In this way, RNA is encapsulated within liposomes. Cationic lipids such as, DOTMA (1,2-di-*O*-octadecenyl-3-trimethylammonium-propane) and DOTAP (1,2-dioleoyl-3-trimethylammonium-propane) readily form complexes with negatively charged RNA. The use of cholesterol modified lipid makes the resulting complex more stable and improves transfection. Hattori et al. (2015) demonstrated the use of cationic cholesterol modified liposome to deliver siRNA into mouse liver. Subsequently, Kranz et al. (2016) demonstrated the use of liposomes for intravenous delivery of mRNA vaccines encoding four tumor antigens NY-ESO-1, MAGE-A3, tyrosinase and TPTE targeting dendritic cells (DCs) in patients with advanced malignant melanoma, effectively expressing the antigens in spleen cells. However, the liposome delivery systems have flaws; (1) liposomes are less stable, and may fuse or leak RNA resulting in low efficiency of delivery; (2) they entrap less RNA; (3) they can be harmful if oxidized; and (4) are not consistent in size. The heterogeneity of the particles increases batch to batch variability (Laouini et al., 2012; Sercombe et al., 2015; Shin et al., 2018).

Some of the aforementioned hurdles have been addressed with advances in the surface modifications of liposomes. Lipid nanoparticles (LNP) made of cationic and other lipids, cholesterol, and polyethylene glycol (PEG) with a hydrophilic inner core retain the capacity to carry anionic RNA, protect it from degradation and prolong its circulation. An example of the efficacy of the LNP platform is patisiran (Onpattro, Alnylam Pharmaceuticals, FDA approval August 2018), the siRNA-based therapeutic against hereditary transthyretin-mediated amyloidosis discussed above. In patisiran, the dsRNA is encapsulated inside four lipid excipients (Kristen et al., 2018): DSPC [1,2-distearoyl-sn-glycero-3-phosphocholine], cholesterol (DLin-MC3-DMA) [(6Z,9Z,28Z,31Z)-heptatriaconta-6,9,28, 31-tetraen-19-yl-4-(dimethylamino) butanoate] and PEG2000-C-DMG [α-(3′-{[1,2-di(myristyloxy)proponoxy] carbonyl-amino}propyl)-ω-methoxy, polyoxyethylene] (Zhang et al., 2020). The PEG functionalization is crucial for this platform as it allows enough circulation time for the drug to be localized in the liver.

Another example is PEG-liposome mRNA vaccines developed by BioNTech and Moderna for the treatment of COVID-19. As described above, these vaccines encode the prefusion-stabilized SARS-CoV-2 spike protein. Minor concerns have been raised as the proprietary lipid is cleared slowly from target tissues and studies are ongoing if accumulation of this lipid could represent a safety concern. The promising results have been seen in non-human primates and human, and, at the time of the writing of this manuscript, this formulation has been approved by FDA for emergency use. Another concern was raised by the rare anaphylactic reactions to the RNA vaccines, which have been attributed to the PEG component of the LNPs (Castells and Phillips, 2021).

**FIGURE 9:**
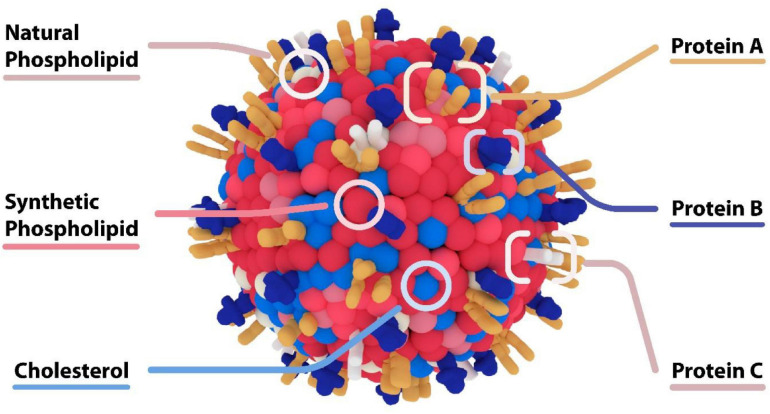
Biomimetic Nanoparticles – “Leukosome” Technology. Biomimetic nanoparticles developed by Molinaro et al. (2016) “Leukosomes.” The composition of these particles consists of a liposome together with proteins derived from leukocytes. These particles preferentially adhere to activated endothelium at sites of inflammation, and have intrinsic anti-inflammatory properties.

Beyond PEG-functionalized liposomes, more sophisticated surface modalities have emerged to create “biomimetic” nanoparticles that functionalized membrane proteins on the surface of nanoparticles (see Figure 9). We have generated “leukosomes” by incorporating leukocyte membrane proteins into the surface of liposomes (Molinaro et al., 2016). These particles have the virtue of localizing at sites of inflammation, binding to the activated endothelium via endothelial adhesion molecules such as LFA-1 and CD-45 (Molinaro et al., 2016). Furthermore, this biomimetic platform has shown intrinsic anti-inflammatory effects upon endothelium through its interaction with macrophages. In a lipopolysaccharide (LPS) induced murine model of sepsis, administration of biomimetic nanoparticles derived from macrophages decreased pro-inflammatory genes (IL-6, IL-1b, and TNF-α), and increased anti-inflammatory genes (IL-10 and TGF-β) (Molinaro et al., 2019). The intrinsic anti-inflammatory effects of these biomimetic leukosomes have been demonstrated in other disease models including inflammatory bowel disease (IBD) (Corbo et al., 2017), atherosclerosis (Martinez et al., 2018; Boada et al., 2020), and cancer (Martinez et al., 2018). Most recently, we have shown the capability of leukosomes to home to sites of vascular inflammation in the apo E deficient hypercholesterolemic mouse. In this model, the elevated levels of cholesterol cause the accumulation of lipid and macrophages in the aorta. Leukosomes were more efficient than standard LNPs at delivering rapamycin to the aorta, where the rapamycin inhibited macrophage proliferation and generation of inflammatory cytokines (Boada et al., 2020). While significant work remains to be done testing the efficacy of this platform in larger animal models, there is great promise for biomimetic nanoparticles to create new mRNA therapeutics that has the potential to selectively target all the inflammatory-based conditions.

### Polymer Nanomaterials

Polymer nanomaterials normally refer to synthetic compounds made of a handful of base units that come together to form complex structures. These materials usually include synthetic polymers such as PLGA [ploy(lactic-co-glycolic acid)], PLA (polylactic acid), chitosan, gelatin, polycaprolactone, and poly-alkyl-cyanoacrylates. These materials have the virtue of a long shelf life; the ability to encapsulate hydrophilic and hydrophobic compounds and proteins; and the capability for tuned delivery of therapeutic compounds (Devulapally et al., 2016; Amini et al., 2017). The flexibility inherent in polymeric nanomaterials is due to the fact that small chemical modifications of the basic polymeric units permits exquisite control over release profile. Polymers can be synthesized to create injectable nanoparticles that can be delivered as intravenous injections or administered as intramuscular, subdermal or intraperitoneal drug depots that degrade over a period of months or weeks (Molina et al., 2015).

The ease of encapsulation extends to nucleic acids. Cationic hydrophilic polymers with hydrophobic modification can self-assemble in aqueous phase to encapsulate RNA. An example of this was employed by Zhao et al. (2016), who developed cationic polyethyleneimine-stearic acid (PSA) copolymer to deliver HIV-1 gag encoding mRNA to dendritic cells (DC2.4 cells) and BALB/c mice. The optimum mass ratio of PSA: mRNA was 4:1, as assessed by transfection efficiency. They injected 6–8 weeks old female mice (BALB/c) subcutaneously with HIV-1 gag mRNA encapsulated in PSA and detected levels of anti-HIV-1 gag specific antibodies. They also demonstrated HIV gag specific CD8+ and CD4+ T cell responses after immunization with PSA/mRNA vaccine (Zhao et al., 2016). McCullough et al. (2014) used chitosan nanoparticles to deliver self-amplifying replicon RNA encoding influenza virus hemagglutinin and nucleoprotein to dendritic cells in mice and rabbit (McCullough et al., 2014). Andreozzi et al. (2017) explored the pH sensitivity of their nucleic acids carrier to trigger endosomal delivery and found that the carrier is stable at narrow pH range (7–9). They used poly(allylamine) phosphate supramolecular nanocarriers to deliver green fluorescence protein (GFP) siRNA in A549 cells to effectively silence the expression of GFP protein.

Consideration must also be given to the intrinsic tendency of polymers to induce inflammation within the immediate microenvironment, due either to lack of degradation such as in the case of PLA or through byproducts. In the case of PLGA, degradation into its basic monomers, lactic acid and glycolic acid leads to a decrease in pH at sites of degradation, promoting inflammation (Kumari et al., 2010). This is not an insurmountable problem. One popular and practical solution has been the use of lipid-polymer hybrids pioneered by Zhang et al. (2008). This type of material, a nanoparticle with a lipid surface and a polymer core, combines the best of both worlds and provides an exquisite control over release profile from its polymer core and lipid surface that better resembles the cell membrane, with possibility for modification that can further enhance targeting toward diseased tissues. Application of this material to deliver RNA therapeutics is promising based on pre-clinical results with xenograft tumors (Shi et al., 2011). The combination of lipid and polymer permits combination therapy. In a landmark study, (Desai et al., 2013) delivered anti-TNFα siRNA (siTNFα) together with capsaicin, in a psoriatic plaque murine model. Their polymer nanoparticle was able to penetrate the cutaneous barrier to deliver the therapeutic interference RNA. Given these pre-clinical results the use of polymer-based materials is extremely promising for delivery of RNA therapeutics (Hadinoto et al., 2013).

### Silica Nanoparticles

Mesoporous silica nanoparticles (MSNPs) have gained more attention for their therapeutic applications. The nanoparticles consist of an amorphous silica (silicon dioxide) matrix with ordered porosity in the mesoporous range. The peculiar feature of this nanoparticle includes large surface areas with large pore volumes, ease of modification, and established silanol chemistry. The surface of the nanoparticles can be modified by positively charged moieties to transport negatively charged RNA. The MSNPs pore sizes are tunable with a broad range and can be quite uniform. The particles have high loading capacity for nucleic acids and efficient delivery. RNA can be loaded inside pores for their transport and the surface can be modified with cancer specific ligands and antibodies to deliver the RNA to the target site. Particle aggregation is a hurdle that must be overcome so that the product is safe for injection. Aggregation can cause thrombosis or induce tissue injury. However, PEGylation of the surface of the nanoparticles greatly decreases aggregation and tissue damage. These nanoparticles degrade into non-toxic products and are safely excreted (Möller et al., 2016; Paris and Vallet-Regí, 2020). Meka et al. (2016) developed the MSNPs with large pore and bicontinuous cubic mesostructure to deliver siRNA in human colon cancer cells (HCT116). They demonstrated the high efficiency of the silica particles in drug delivery and suppression of the tumor proteins in their work (Meka et al., 2016). Lee et al. (2018) developed hybrid particles with a positive charge structure with large pores to load anionic siRNA drug (knockdown B cell lymphoma 2, Bcl-2) and a negatively charged structure with small pores to load the anticancer drug, doxorubicin. They used their hybrid particles to deliver siRNA and doxorubicin to HeLa cells. Their work demonstrated the flexibility of this platform to provide dual pore hybrid silica nanoparticles to treat cancer with combination of both genetic and chemotherapeutic drugs simultaneously (Lee et al., 2018).

### Carbon and Gold Nanomaterials

Gold nanoparticles, quantum dots, nanographene oxide, carbon nanotubes are each synthesized nanostructures that have the capacity to harbor RNA, protecting it from degradation and delivering it to the targeted disease site. Zheng et al. (2012) used gold nanoparticles for topical application of therapeutic nucleic acids (siRNA, knockdown EGFR) in mouse and human skin (Zheng et al., 2012). Li et al. (2011) used quantum dots-siRNA complexes to suppress a target gene (HPV18 E6 gene) in HeLa cells. Yang et al. (2014) utilized nanographene oxide modified with gadolinium(Gd-NGO) to deliver a small molecule anti-cancer drug, epirubicin together with Let-7g miRNA (tumor suppressors, decrease expression of the Ras oncogene family) to image and treat glioblastoma in mice (Yang et al., 2014). Kam et al. (2005) observed that functionalized carbon nanotubes could deliver siRNA against lamin A/C to suppress the expression of this protein in HeLa cells.

### *N*-Acetylgalactosamine (GalNAc)

GalNAc is a trivalent ligand that binds to asialoglycoprotein (ASGPR) receptors in hepatocytes. Clinical studies suggest that GalNAc conjugated siRNAs are very efficient to knockdown gene expression in the liver. Zimmermann et al. (2017) performed the first clinical trial of GalNAc conjugated siRNA drugs (Revisuran) to treat transthyretin-mediated amyloidosis (ATTR) (Zimmermann et al., 2017). However, an imbalance in deaths of the Revisuran siRNA treated patients in phase III clinical trials terminated the study of this drug. Givosiran (Givlaari, developed by Alnylam Pharmaceuticals) is the world’s first ever GalNAc conjugated siRNA drug approved by FDA (November, 2019) (Alnylam, 2020b). As discussed above, Givosiran is used to prevent acute attacks of hepatic porphyria by silencing the expression of aminolevulinate synthase 1 mRNA in liver. This has the effect of reducing neurotoxic levels of aminolevulinic acid and porphobilinogen that can induce seizures, paralysis, respiratory failure, neurological damage and death (Balwani et al., 2020; Nature Biotechnology, 2020). There are few more GalNAc conjugated siRNA drugs (Fitusuran, Lumasiran, Vutrisiran, and Inclisiran) in Phase 3 clinical trials from Alnylam Pharmaceuticals (Alnylam, 2020a).

## Hospital-Based RNA Therapeutics

We are witnessing the dawn of a new era of biopharmacotherapy. The promise of mRNA therapeutics has galvanized the pharmaceutical companies and investment sector, as evidenced by the current market caps of Biontech ($22.4B), Curevac ($18.6B), and Moderna ($52B), as well as the rapid proliferation of biotech start-ups in this field (FierceBiotech, 2020). Each of the three major companies has dozens of new mRNA therapeutics under development, some of which have been mentioned in this review. Inherent advantages of mRNA therapeutics have spurred this unprecedented investment. High-purity RNA constructs can be generated much faster than traditional small molecule drugs or recombinant proteins, and at lower costs; the manufacturing process is adaptable to any RNA sequence allowing for personalized RNA therapeutics; moreover, RNA has a superior safety profile and simpler regulatory roadmap compared to DNA-based gene therapy because it doesn’t integrate into the host genome (Sahin et al., 2014; Karikó, 2019). The rapid growth of mRNA therapeutics has been made possible by recent advances which have overcome key obstacles such as innate immune activation, RNA stability, and delivery.

**FIGURE 10:**
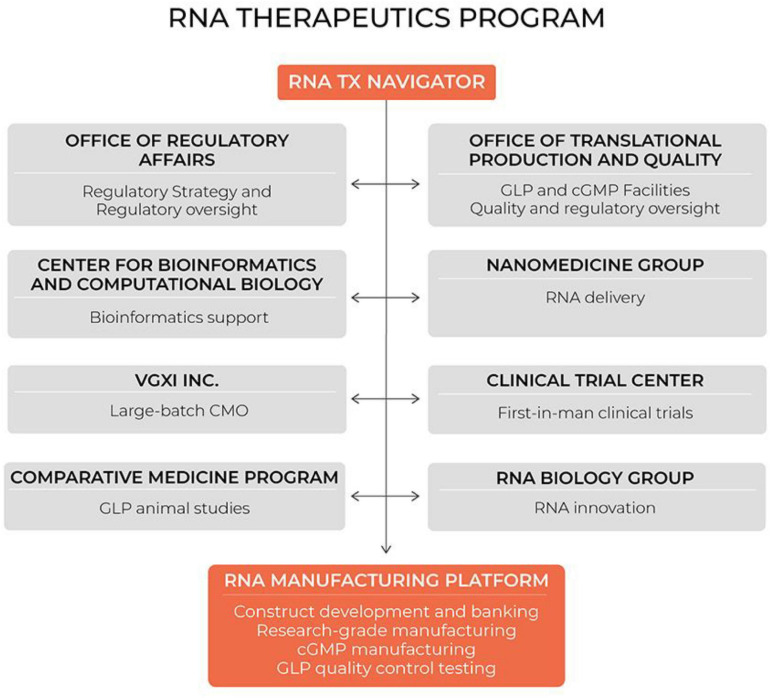
Hospital-based RNA therapeutics (TX) program in Houston Methodist. CMO, contract manufacturing organization; GLP, good laboratory practice; cGMP, current good manufacturing practice.

Notably, mRNA therapeutics are a disruptive therapeutic technology, as small biotech startups, as well as academic groups, can rapidly develop new and personalized mRNA constructs. Our group has long-standing expertise in designing and manufacturing RNA therapeutics for the scientific community. Initially we were funded by the NHLBI to serve as an mRNA core for stem cell investigators in the Progenitor Cell Biology Consortium. Subsequently, the Cancer Prevention and Research Institute of Texas funded us to address an unmet need for cancer researchers in Texas who use mRNA as a research tool or a therapeutic. The demand for our services has increased substantially within the last 5 years, while we strengthened our expertise through extensive collaboration with cancer biologists, immunologists, RNA biologists, bioinformaticians, and nanomedicine scientists. During this time we established our hospital-based cGMP-compliant manufacturing capability and quality control methods (see Figure 10). We licensed our proprietary processes to VGXI Inc, a local company with large-batch manufacturing capabilities for DNA-based gene therapies. With this collaboration, we built a seamless transition for academic groups and small companies to go from pre-clinical development and first-in-man clinical trials supported by our hospital-based program, to late-stage clinical trials and commercialization supported by our industry partner VGXI, Inc.

During these years we learned that many small biotech companies and academic groups, who have innovative ideas for disruptive RNA therapeutics, lack key competencies to reach the clinic, such as manufacturing capabilities, delivery technologies, or regulatory expertise, while our group has been operating in the environment established to accelerate novel therapeutics from conception to the clinic. Houston Methodist Research Institute is adjacent to the Houston Methodist Hospital, which is ranked by US News and World Report as the No. 1 hospital in Texas. The Institute was established with the focus on clinical translation. As such, the Institute has the infrastructure necessary to support pre-clinical and clinical development of novel therapeutics. We have clean rooms and calibrated equipment for cGMP manufacturing of RNA constructs, as well as the equipment to synthesize and analyze lipid nanoparticles for RNA encapsulation. We have an Office of Translational Production and Quality that provides guidance and quality oversight for manufacturing, QC testing and release. In addition, our Office of Regulatory Affairs assists with regulatory roadmaps and translational research planning. Our Comparative Medicine Program supports GLP animal studies for IND applications, while our Cockrell Clinical Trial Center designs and implements first-in-man clinical trials. This infrastructure has facilitated our development of the first fully integrated hospital-based RNA therapeutics program.

Our RNA core is the central pillar, as the RNA core team develops new constructs and manufactures research or clinical grade RNA. The RNA core is complemented by RNA biologists, bioinformaticians, and nanomedicine experts. This program offers a single entry point with consultation to ensure a seamless transition between development stages. This system facilitates the development of RNA therapeutics by academic groups and small biotechs and dramatically shortens the time “from bench to bedside.” Furthermore, it promotes collaboration between our RNA faculty and clinician-scientists at the hospital to identify unmet clinical needs and develop mRNA-based solutions.

The success of hospital-based programs to generate novel nucleic acid drugs has already been demonstrated. For instance, the investment of Nationwide Children’s hospital into cGMP facilities and a critical mass of gene therapy experts led to the creation of transformative gene therapies for lethal childhood diseases: SMA (onasemnogene abeparvovec xioi; Zolgensma, reviewed in DNA therapies) and DMD (golodirsen; Vyondys 53). Golodirsen is a phosphorodiamidate morpholino oligomer that hybridizes to exon 53 of dystrophin pre-mRNA, and restores the mRNA reading frame in patients with confirmed DMD mutations amenable to exon 53 skipping (Frank et al., 2020). Besides golodirsen, the group is currently evaluating another treatment for DMD – a gene transfer of micro-dystrophin delivered by recombinant AAV and driven by a skeletal and cardiac muscle-specific promoter with enhanced cardiac expression (Mendell et al., 2020).

Another example of a successful hospital-based gene therapy program is that of St. Jude Children’s Research Hospital. As described above, they developed and generated material in house for first-in-man studies of a lentiviral gene therapy for X-linked SCID. Their lentiviral vector encoding *IL2RG* was designed to include insulators to block activation of genes adjacent to where *IL2RG* is inserted into the patients’ DNA. The goal was to prevent the gene therapy from inadvertently causing leukemia by switching on an oncogene in the patient’s blood stem cells. Vector production and gene therapy treatment were streamlined using a stable producer cell line and cryopreservation. Combined with low-exposure, targeted busulfan conditioning in infants with newly diagnosed SCID-X1, the therapy had only low-grade adverse effects and resulted in multilineage engraftment of transduced cells, reconstitution of functional T cells and B cells, and normalization of NK-cell counts during a median follow-up of 16 months (Mamcarz et al., 2019).

In addition to accelerating clinical translation of novel therapeutics, hospital-based programs facilitate development and implementation of personalized medicines, when relatively small quantities of a drug are required in treating a single patient. While manufacturing of the drug at such scale is rarely financially justified for a big pharma, it is quite reasonable for a hospital, can be done within a hospital’s cGMP facilities and then quickly delivered to the patient. An outstanding example of patient-customized treatment was recently demonstrated by Boston Children’s Hospital. In patient presenting with symptoms diagnostic of Batten’s disease, genetic testing for known mutations were not observed. Whole-genome sequencing revealed a previously unknown insertion that altered splicing of the MFSD8 gene and led to premature translational termination. Knowing this, the investigators designed a 22-mer ASO, milasen, with the same backbone and sugar chemistry modifications as nusinersen (reviewed in ASO section) to correct the misplicing and restore MFSD8 expression. Soon after pre-clinical studies to confirm efficacy and assess toxicity studies, the drug was administered to the patient with clinical improvement observed (Kim et al., 2019). Notably, the first injection of milasen was done within 1 year after first contact with the patient, a testament to the speed of this hospital-based gene therapy platform.

## Conclusion and Future Perspectives

RNA Therapeutics is a rapidly emerging field of biotherapeutics. These therapies are based upon powerful and versatile platform which has nearly unlimited capacity to address unmet clinical needs. RNA Therapeutics are destined to change the standard of care for many diseases. The number of RNA drugs under development, and in clinical trials, is growing rapidly. The rapid growth of RNA therapeutics has been due to solving the problems of stability, delivery and immunogenicity. Whereas there is room for further improvement and innovation in each of these areas, the solutions have advanced to the point that RNA Therapeutics are now feasible. Although several dominating players in the RNA biopharma sector have emerged, new small biotech startups as well as academic groups with transformative ideas are propagating. In addition, hospital-based RNA therapeutics programs will facilitate RNA-based drug development and accelerate translation of transformative therapies from lab bench to patient’s bedside.

## Author Contributions

TRD: first draft and illustrations. RS, CB, FT, and RIP: subsequent revisions of manuscript. JPC: revisions of the manuscript and funding of effort. All authors contributed to the article and approved the submitted version.

## Conflict of Interest

Houston Methodist Hospital has been assigned intellectual property related to the synthesis, purification, validation, and delivery of nucleic acid therapeutics. JPC is an inventor on issued patents related to mRNA telomerase therapy, which have been assigned to Stanford University and licensed to his company. The remaining authors declare that the research was conducted in the absence of any commercial or financial relationships that could be construed as a potential conflict of interest.
